# *Xenorhabdus khoisanae* SB10 produces Lys-rich PAX lipopeptides and a Xenocoumacin in its antimicrobial complex

**DOI:** 10.1186/s12866-019-1503-x

**Published:** 2019-06-13

**Authors:** J. Dreyer, M. Rautenbach, E. Booysen, A. D. van Staden, S. M. Deane, L. M. T. Dicks

**Affiliations:** 1 Department of Microbiology, Stellenbosch University, Private Bag X1, Matieland, 7602 South Africa; 2 BIOPEP Peptide Group, Department of Biochemistry, Stellenbosch University, Private Bag X1, Matieland, 7602 South Africa

**Keywords:** *Xenorhabdus khoisanae*, Antimicrobial complex, Lys-rich PAX lipopeptides, Xenocoumacin, Mass spectrometric analysis

## Abstract

**Background:**

*Xenorhabdus* spp. live in close symbiosis with nematodes of the *Steinernema* genus. *Steinernema* nematodes infect an insect larva and release their symbionts into the haemocoel of the insect. Once released into the haemocoel, the bacteria produce bioactive compounds to create a semi-exclusive environment by inhibiting the growth of bacteria, yeasts and molds. The antimicrobial compounds thus far identified are xenocoumacins, xenortides, xenorhabdins, indole derivatives, xenoamicins, bicornutin and a number of antimicrobial peptides. The latter may be linear peptides such as the bacteriocins xenocin and xenorhabdicin, rhabdopeptides and cabanillasin, or cyclic, such as PAX lipopeptides, taxlllaids, xenobactin and szentiamide. Thus far, production of antimicrobial compounds have been reported for *Xenorhabdus nematophila, Xenorhabdus budapestensis, Xenorhabdus cabanillasii*, *Xenorhabdus kozodoii*, *Xenorhabdus szentirmaii*, *Xenorhabdus doucetiae*, *Xenorhabdus mauleonii*, *Xenorhabdus indica* and *Xenorhabdus bovienii*. Here we describe, for the first time, PAX lipopeptides and xenocoumacin 2 produced by *Xenorhabdus khoisanae*. These compounds were identified using ultraperformance liquid chromatography, linked to high resolution electrospray ionisation mass spectrometry and tandem mass spectrometry.

**Results:**

Cell-free supernatants of *X. khoisanae* SB10 were heat stable and active against *Bacillus subtilis* subsp. *subtilis*, *Escherichia coli* and *Candida albicans*. Five lysine-rich lipopeptides from the PAX group were identified in HPLC fractions, with PAX1’ and PAX7 present in the highest concentrations. Three novel PAX7 peptides with putative enoyl modifications and two linear analogues of PAX1’ were also detected. A small antibiotic compound, yellow in colour and λ_max_ of 314 nm, was recovered from the HPLC fractions and identified as xenocoumacin 2. The PAX lipopeptides and xenocoumacin 2 correlated with the genes and gene clusters in the genome of *X. khoisanae* SB10.

**Conclusion:**

With UPLC-MS and MS^e^ analyses of compounds in the antimicrobial complex of *X. khoisanae* SB10, a number of PAX peptides and a xenocoumacin were identified. The combination of pure PAX1’ peptide with xenocoumacin 2 resulted in high antimicrobial activity. Many of the fractions did, however, contain labile compounds and some fractions were difficult to resolve. It is thus possible that strain SB10 may produce more antimicrobial compounds than reported here, as suggested by the APE Ec biosynthetic complex. Further research is required to develop these broad-spectrum antimicrobial compounds into drugs that may be used in the fight against microbial infections.

**Electronic supplementary material:**

The online version of this article (10.1186/s12866-019-1503-x) contains supplementary material, which is available to authorized users.

## Background

*Xenorhabdus* bacteria are in a species-specific association with *Steinernema* nematodes, i.e. a specific *Steinernema* sp. is associated with a specific *Xenorhabdus* sp. [1]. At the beginning of the *Xenorhabdus-Steinernema* life cycle, nematodes in the infective juvenile phase infect the insect host by entering the mouth, anus or respiratory spiracles [2]. Once inside the insect, the nematodes release the symbiotic bacteria by defecation. *Steinernema* nematodes produce proteins that suppress the insect’s immune response, which allows *Xenorhabdus* to multiply [3]. The release of exoenzymes and toxins by both mutualists leads to septicaemia and the insect dies within 24 to 48 h after infection [4–6]. Nematodes reproduce sexually by going through phases J1 to J4 until resources are depleted, after which they return to the infective juvenile state, acquire symbionts and leave the cadaver in search of a new host.

During the nematode life cycle, *Xenorhabdus* spp. produce various compounds to create a semi-exclusive environment and prevent colonisation of the host (insect) by other microorganisms [7]. Dutky [8] was the first to suggest that *Xenorhabdus* produce antimicrobial compounds. However, interest in these antimicrobial compounds only gained momentum 22 years later [9]. Numerous bioactive compounds have since then been detected in the cell-free supernatants of *Xenorhabdus* spp., including broad-spectrum compounds with activity against bacteria, fungi, insects, nematodes, protists and cancer cells. These compounds range from being small antibiotics, such as xenocoumacins [10], xenortides [11], xenorhabdins [12] and indole derivatives [13], to larger, more complex, compounds such as xenoamicins [14], bicornutin [15] and various antimicrobial peptides (AMPs). Some AMPs are cyclic, such as PAX (peptide-antimicrobial-*Xenorhabdus*) lipopeptides [16], taxlllaids [17], xenobactin [18] and szentiamide [19], while others are linear, such as rhabdopeptides [20], cabanillasin [21] and bacteriocins. Thus far, xenocin and xenorhabdicin, produced by *Xenorhabdus nematophila* [22, 23], are the only bacteriocins characterised to structural level. Bacteriocins have also been isolated from *Xenorhabdus bovienii* and *Xenorhabdus beddingii,* but they have not been fully characterised [24].

Gualtieri and co-workers [16] identified five lysine-rich PAX peptides from *X. nematophila* F1/1 with strong antifungal activity against *Fusarium oxysporum* and several plant pathogenic fungi. Fuchs et al. [25] identified another 13 lysine-rich PAX peptides produced by *X. nematophila* HGB081. The PAX peptides have an identical backbone structure, with cyclization occurring between the ε-amino group of Lys^3^ and the C-terminus. According to NMR studies, the fatty acids in PAX peptides from *X. nematophila* F1/1 are iso-branched [26], whilst those of *X. nematophila* HGB081 are straight-chain fatty acids [25]. Three NRPS genes (*paxA*, *paxB* and *paxC*) encode biosynthesis of the PAX peptides [25]. PAX peptides have antifungal and antibacterial activity, but they are not cytotoxic towards insects [25, 26].

The antimicrobial compounds are generally used in combination with the associated nematodes as biological control agents in the agricultural industry [26–30]. A number of antimicrobial compounds produced by *Xenorhabdus* spp. have been patented [31–37]. For the first time we describe a number of PAX lipopeptides from the A-group and xenocoumacin that are co-produced by *Xenorhabdus khoisanae* SB10.

## Results and discussion

**Fig. 1:**
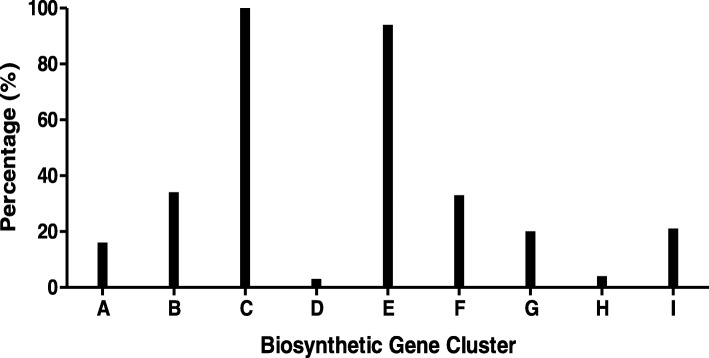
AntiSMASH results presenting the similarity between the genome of *X. khoisanae* SB10 and biosynthetic gene clusters of Thiomarinol (A), Zeamine (B), Xenocoumacin (C), Lysobactin (D), APE Ec (E), Xenoamicin (F), Safracin (G), Taxlllaid (H) and Acinetobactin (I)

*Xenorhabdus* bacteria are known to produce various antimicrobial compounds, but it is a highly neglected antimicrobial source that has not been exploited to its full potential. Although many genes relevant to antimicrobial compound biosynthesis have been identified in *Xenorhabdus* spp., the isolation, purification, identification and characterisation of antimicrobial compounds have not been done for all species belonging to this genus. Our antiSMASH [38] analysis of the genome of *X. khoisanae* SB10 revealed the presence of the xenocoumacin biosynthetic gene cluster and an APE Ec gene cluster (Fig. 1). The APE Ec gene cluster is widely distributed amongst prokaryotes and is related to secondary metabolites such as aryl polyenes [39]. Identification of the four modules coding for the PAX synthetase complex in the genome of *X. khoisanae* SB10 was accomplished by using tblastx (Table 1). This study, therefore, focussed on the first isolation and confirmation that specific antimicrobial compounds are indeed produced by *X. khoisanae* SB10. The following section describes the purification and characterisation of a selection of antimicrobial compounds from *X. khoisanae* SB10 cultures. A summary of the UPLC-MS data and identification of the compounds in the antimicrobial fractions are presented in Table 2. Detailed analysis utilising ESMS, UPLC-MS and MS^e^ data on these fractions can be found in the Additional file 1 section.

**Table 1:** Identification of the four modules coding for the PAX synthetase complex in the genome of *X. khoisanae* SB10

Gene	Protein product	Synthetase Protein	Presumed function	Position on Chromosome	Identity (%)	Positivity (%)	Origin	Accession number
*xcn1_2784*	PaxT	XpsD	ABC-transporter	Node 4–50,691 to 52,328	76 (416/549)	88 (487/549)	*X. bovienii* SS-2004	CBJ81280.1
*xcn1_2783*	PaxA	XpsA	NRPS	Node 4–52,788 to 56,036	62 (678/1098)	76 (836/1098)	*X. bovienii* SS-2004	CBJ81279.1
*xcn1_2782*	PaxB	XpsB	NRPS	Node 4–66,118 to 76,893	63 (2265/3614)	76 (2747/3614)	*X. bovienii* SS-2004	CBJ81277.1
*xcn1_2781*	PaxC	XpsB	NRPS	Node 4–56,079 to 66,107	69 (2307/3356)	81 (2733/3356)	*X. bovienii* SS-2004	CBJ81278.1

**Table 2:** Summary of the antimicrobial compounds in the three main absorbing fractions that were identified using UPLC-MS and UPLC-MS^e^. PAX peptide identities and names are from Fuchs et al. [32]

Fraction	UPLC Rt (min)	*m/z* of major [M + H]^+^	Compound *M*_r_^a^	Theoretical *M*_r_^b^	Mass error (ppm)^c^	Proposed compound identity
B	4.27	407.2182	406.2104	406.2104	0.0	Xenocoumacin 2 (C_21_H_30_N_2_O_6_)
	2.93	1052.7948	1051.7870	1051.7845	2.4	PAX1’ (C_14_H_27_O_2_)GK_6_
C	3.18	1078.8119	1077.8041	1077.8001	3.7	PAX7E1* (C_16_H_29_O_2_)GK_6_
	3.42	1070.8091	1069.7921	1069.7951	−2.8	PAX1L* (C_14_H_29_O_3_)GK_6_
D	3.29	1066.8097	1065.7965	1065.8001	−3.4	PAX3’ (C_15_H_29_O_2_)GK_6_
F	3.47	1054.8115	1053.8012	1053.8001	1.0	PAX1L-DH* (C_14_H_29_O_2_)GK_6_
	3.32, 3.47^#^	1080.8286	1079.8191	1079.8158	3.1	PAX7 (C_16_H_31_O_2_)GK_6_
	3.71	1078.8096	1077.8002	1077.8001	0.1	PAX7E2* (C_16_H_29_O_2_)GK_6_
	3.88	1078.8070	1077.7979	1077.8001	−2.0	PAX7E3* (C_16_H_29_O_2_)GK_6_
G	2.51	1050.7723	1049.7623	1049.7688	−6.2	PAX5 (C_14_H_25_O_2_)GK_6_
	2.90	1052.7936	1051.7821	1051.7845	2.3	PAX1’ (C_14_H_27_O_2_)GK_6_
	3.57	1080.8280	1079.8161	1079.8158	0.3	PAX7 (C_16_H_31_O_2_)GK_6_
	3.83	1106.8412	1105.8294	1105.8314	−1.8	PAX8 (C_18_H_33_O_2_)GK_6_

^a^Experimental monoisotopic *M*_r_ of compound was calculated using the TOF transform or MaxEnt3 function in the MassLynx 4.01 software package

^b^Theoretical monoisotopic *M*_r_ of compound was calculated from accurate monoisotopic *M*_r_ of Lys =128.09496 and Gly = 57.02146, and monoisotopic *A*_r_ of O =15.9949146; H=1.0078250, N=14.0030740 and C=12.0000000

^c^Mass error in parts per million (ppm) = 10^6^×{*M*_r_ (theoretical) - *M*_r_ (experimental)}/ *M*_r_ (theoretical)

^#^Early elution of broad peak, fronting and tailing due to aggregation at high concentration

*Putative identification as PAX peptides from peptide moiety fragments and accurate mass determination, E denotes an enoyl group, L denotes linear, DH denotes dehydroxylated, structure of R-group was not elucidated

Refer to Additional file 1 for UPLC-MS, ESMS and MS/MS data on all the compounds

**Fig. 2:**
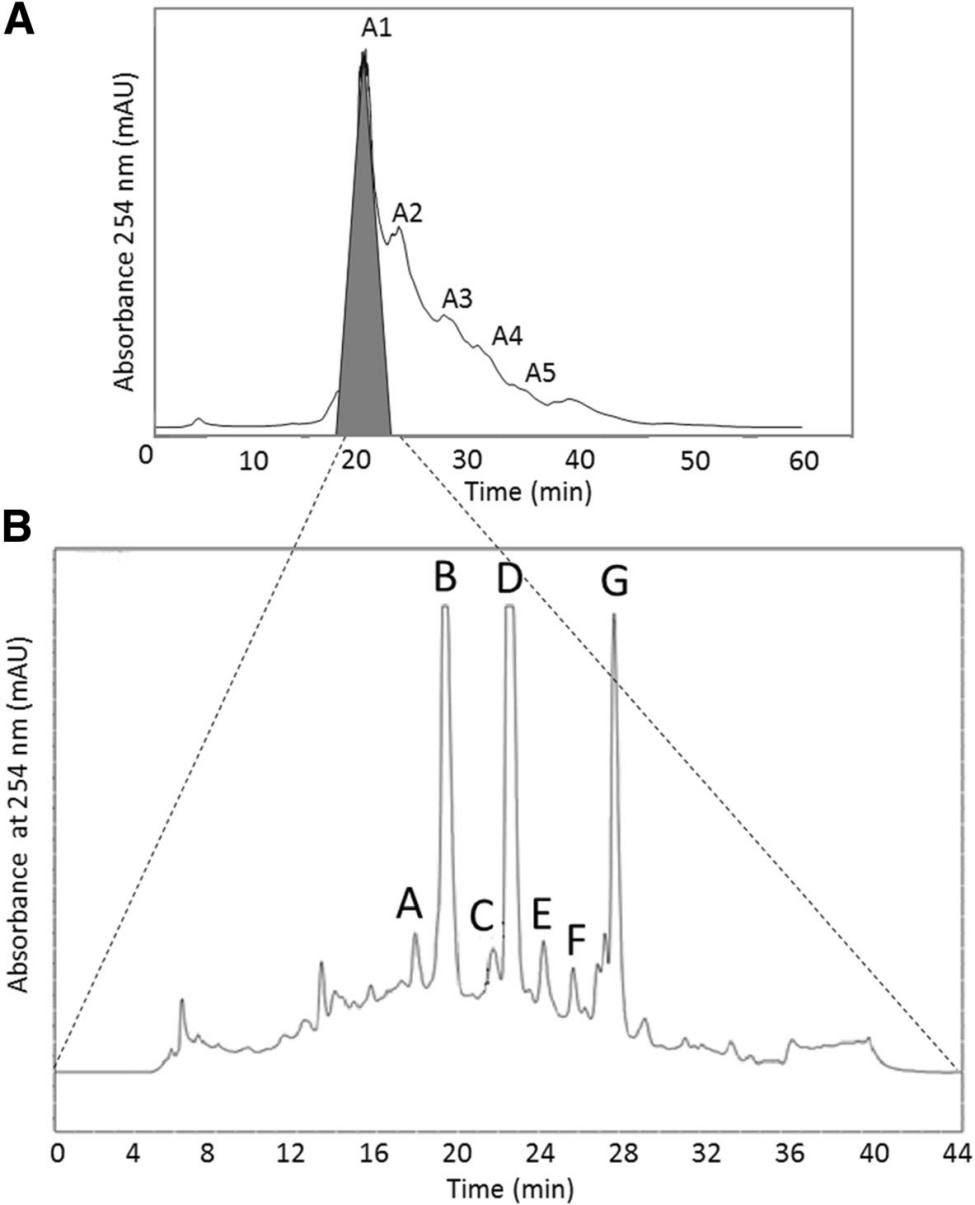
Representative chromatograms depicting the isolation of antimicrobial fractions from the *X. khoisanae* SB10 culture extracts. **a** Separation of SPC active fractions on 15 RPC resin by FPLC, with a linear gradient of 10 to 55% (v/v) acetonitrile containing 0.1% (v/v) TFA. **b** C_18_ HPLC chromatography of the fraction A1 in graph A (FPLC active fraction). A linear gradient from 25 to 45% acetonitrile containing 0.1% TFA was used. The peak fractions denoted A-G in graph B displayed antimicrobial activity

**Fig. 3:**
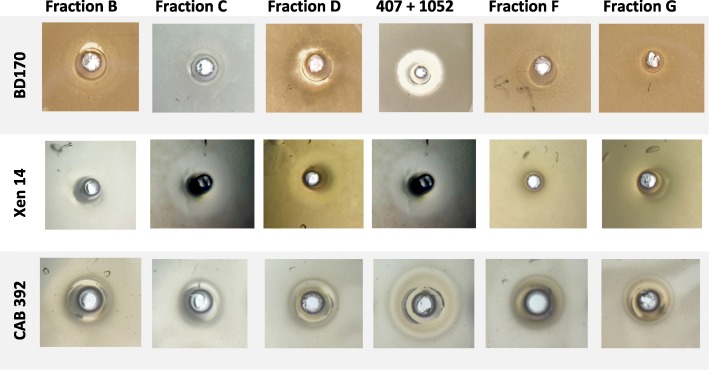
Antimicrobial activity of the factions collected (fractions B, C, D, F and G) and the combined activity of purified xenocoumacin 2 (*m/z* 407) and PAX1’1 (*m/z* 1052) from the compound library of the Department of Microbiology. Growth inhibition is observed as zones surrounding the wells. Activity BD170 = *Bacillus subtilis* subsp. *subtilis*, Xen 14 = *Escherichia coli* and CAB 392 = *Candida albicans*

The first chromatography of the SPC active fractions on 15 RPC resin by FPLC yielded fraction A1 with broad-spectrum antimicrobial activity (Fig. 2a). The SPC A1 fraction was shown to have broad-spectrum activity towards the four target organisms (*B. subtilis*, *E. coli* and *C. albicans*), as well as retaining activity after heating at 121 °C for 20 min (results not shown). The latter result indicated marked heat stability of the antimicrobial compounds and eliminated activity related to labile, or volatile compounds and proteins such as proteases. The A1 fraction was further separated with C_18_-HPLC into seven peaks (Fig. 2b). Fractions B, D and G showed strong UV absorption (Fig. 2b), suggesting that they did not only contain PAX peptides, but also xenocoumacin and breakdown products. Antimicrobial activity was observed in fractions A to G (Fig. 2b) and is shown in Fig. 3. We first focused on the three major UV-absorbing fractions, B, D and G, as well as C and F (Fig. 2b), for further analysis using high resolution UPLC-MS and UPLC-MS^e^ (or MS/MS) analyses (refer to Additional file 1 and Table 2).

**Fig. 4:**
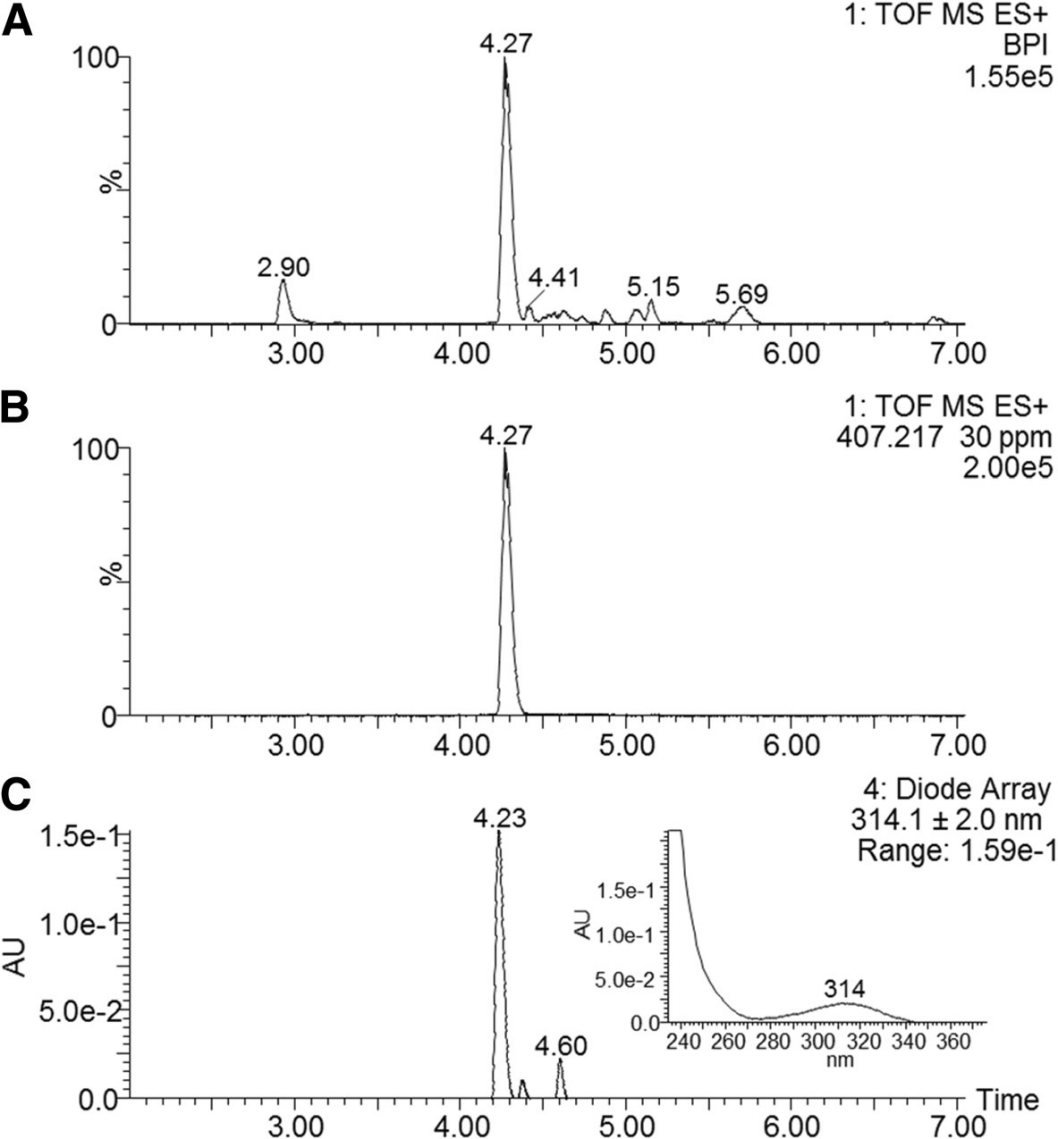
The UPLC profiles of fraction B collected from C_18_-HPLC (refer to Fig. 2 b). The top chromatogram (**a**) shows the base peak intensity mass chromatogram of fraction B and the middle chromatogram (**b**) the mass extracted chromatogram for the molecular ion with *m/z* 407.217 at 30 ppm tolerance. The bottom chromatogram (**c**) shows the spectrophotometric profile at 314 nm and the insert shows the UV spectrum of the peak at 4.23 min

**Fig. 5:**
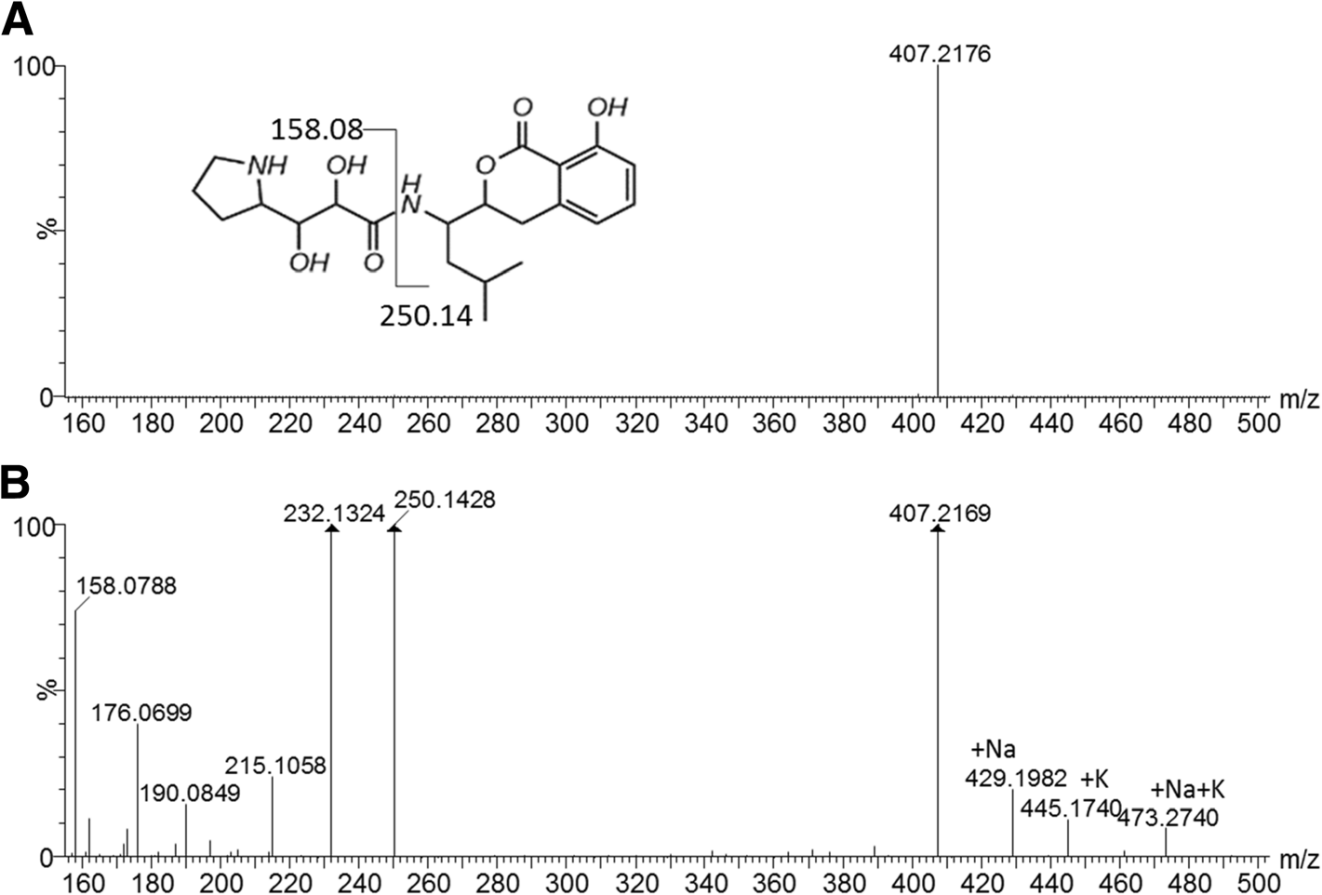
ESI-MS and CID spectra (generated via MS^e^ type analysis) of the main component at 4.23 in fraction B, namely xenocoumacin 2. The component mass spectrum of xenocoumacin 2 (structure insert) is shown in the top spectrum (**a**) and fragmentation product ion spectrum is shown in the bottom spectrum (**b**). The two main fragments are indicated on the xenocoumacin 2 structure. Refer to the text for the discussion of the fragmentation of xenocoumacin 2

The major UPLC-MS peak observed in fraction B contained a small compound with a yellow colour (absorption maximum at 314 nm) and a monoisotopic *M*_r_ of 406.2104 (Fig. 4b and c, insert shows UV-spectrum). The UPLC-MS^e^ analysis of the peak, containing its molecular ion with *m/z* 407.2176 at 4.27 min, yielded six mayor ions (Fig. 5a). The compound was identified as xenocoumacin 2 (expected *m/z* = 407.2182) based on the characteristic fragmentation pattern. The ion with *m/z* 250.1428 (Fig. 5a) represents the benzopyran-1-one fragment (expected *m/z* = 250.1443), while the ion with *m/z* 158.0788 (Fig. 5a) correlates to the remaining fragment with pyrrolidine as R group (expected *m/z* = 158.0817) [37]. Ions with *m/z* 176.0699, 190.0849, 215.1058 and 232.1324 (Fig. 5b) are the hydration and dehydration products of the two main fragments with *m/z* 158.0788 and 250.1428 (Fig. 5b). This identification of a xenocoumacin produced by *X. khoisanae* SB10, was supported by antiSMASH [39] results of 100% similarity between the genome of strain SB10 and the xenocoumacin biosynthetic gene cluster (refer to Fig. 1).

**Fig. 6:**
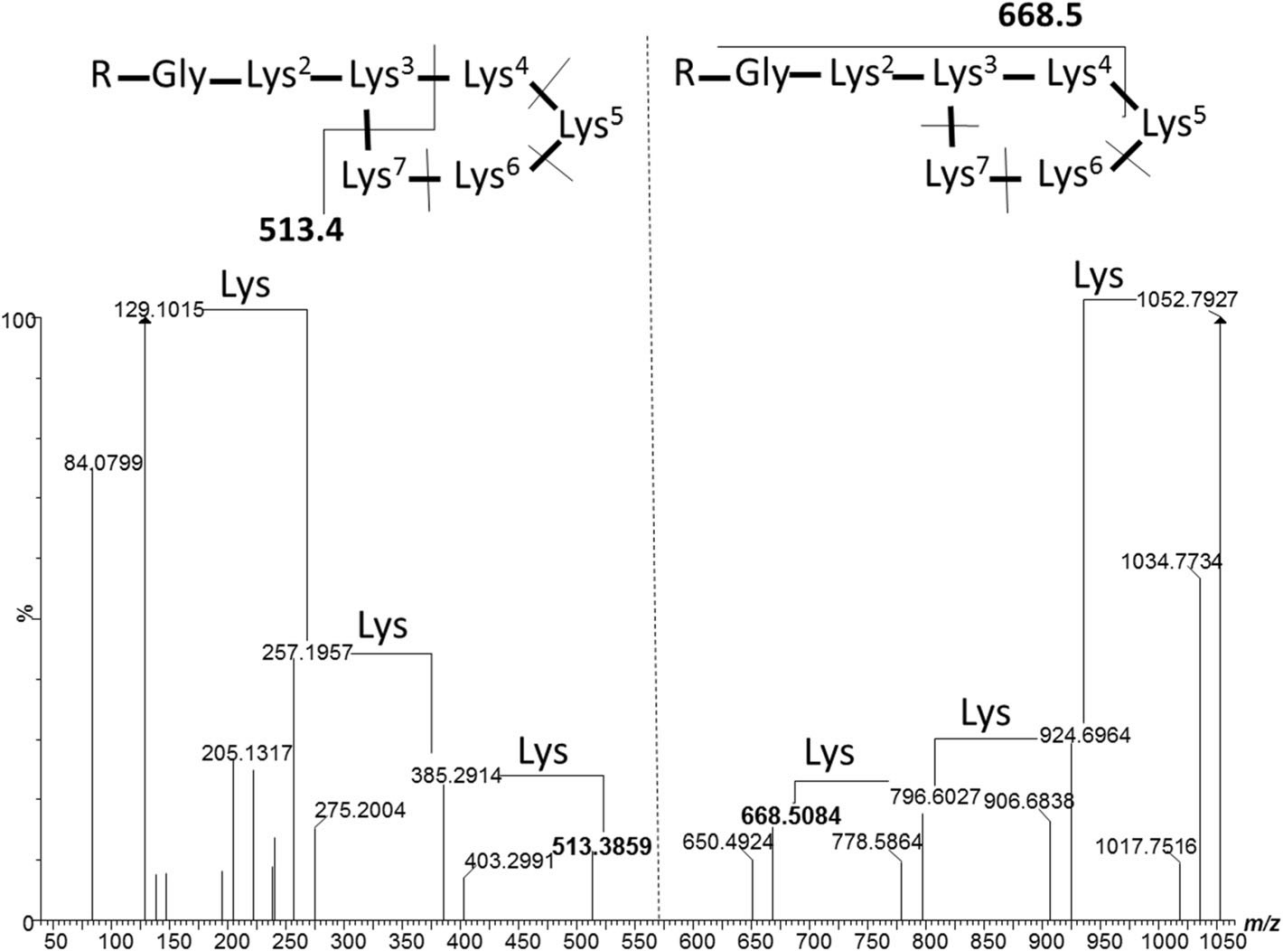
Representative CID spectrum over *m/z* 50–1050 of PAX1’ with intact molecular ion at *m/z* = 1052.79. CID analyses were performed over a CE gradient from 20 to 60 eV at a CV of 15 V. The two structures above the spectrum show the fragments that would lead to the 513.4 and 668.5 product ions and their subsequent fragmentation. The R group for PAX1’ is (3R)-3-hydroxy tetradecanoyl and the side-chain of Lys^3^ links up to the carboxyl group of Lys^7^ to form the ring structure

Fraction B also contained an earlier eluting peak at 2.90 min (refer to Fig. 4a) of a larger compound with *m/z* 1052.7948 (*M*_r_ = 1051.7870). The fragmentation pattern of the ion with *m/z* 1052.7948 showed a neutral loss of 128.09 from the major fragments, which is indicative of the loss of multiple Lys-residues from a peptide chain. The majority of ions with a neutral fragment loss also had resultant dehydration products. This dehydration is the consequence of a fragmentation reaction at the C-terminal of a Lys residue that leads to cyclisation in which the amino group of the Lys side chain participates, similar to fragmentation reactions found for ornithine-containing peptides [40]. Ions with *m/z* 129.1015 and 84.0799 also maps to Lys and its immonium ion. The ion spectrum of *m/z* 1052.7948 and proposed fragmentation pattern is presented in Fig. 6. From this spectrum we were able to map the sequence to a Lys-rich lipopeptide from the PAX peptide group. This particular peptide was identified as PAX1’ with the R group as (3*R*)-3-hydroxytetradecanoyl coupled to Gly-Lys_2_-cyclo(Lys_4_) with an expected *M*_r_ of 1051.7845 (refer to Fig. 6 for structure). Although fraction B did not show high activity against *E. coli* Xen 14, high activity was recorded against all other target strains when purified PAX1’ and xenocoumacin were combined in a 1:1 ratio (Fig. 3). This result warrants further investigation to determine if the activity between PAX peptides and small water soluble xenocoumacins are synergistic.

**Fig. 7:**
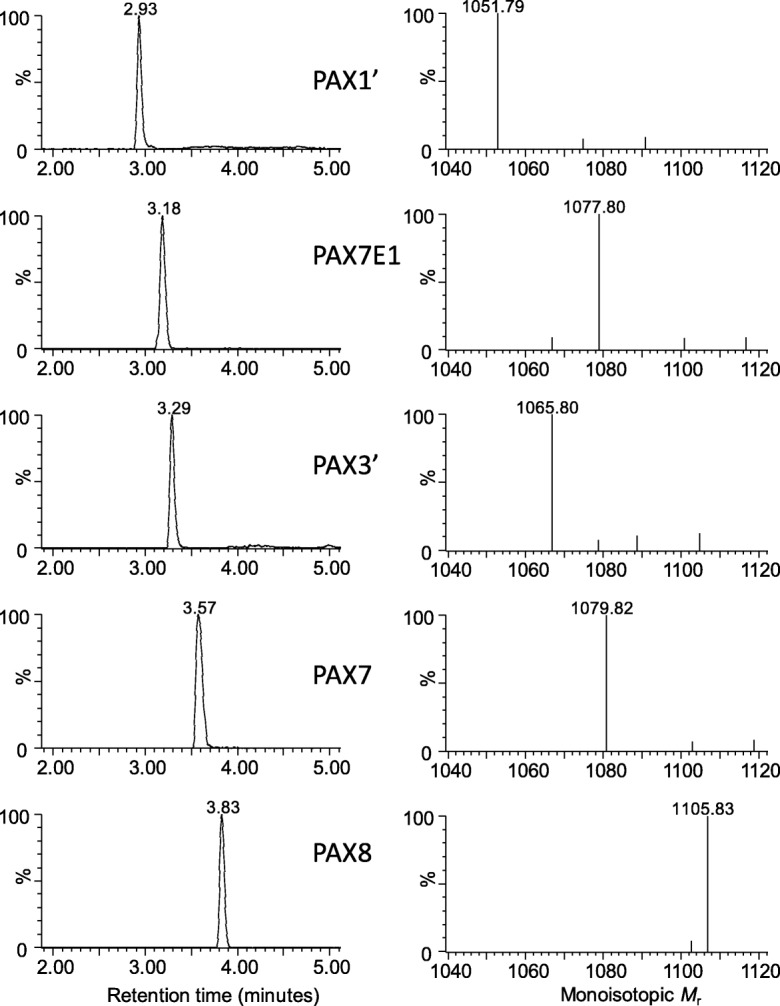
UPLC-MS profiles (left panel) and ESI-MS spectra (right panel) of the five major PAX lipopeptides that were detected in the antimicrobial fractions of the *X. khoisanae* SB10 culture extracts

**Fig. 8:**
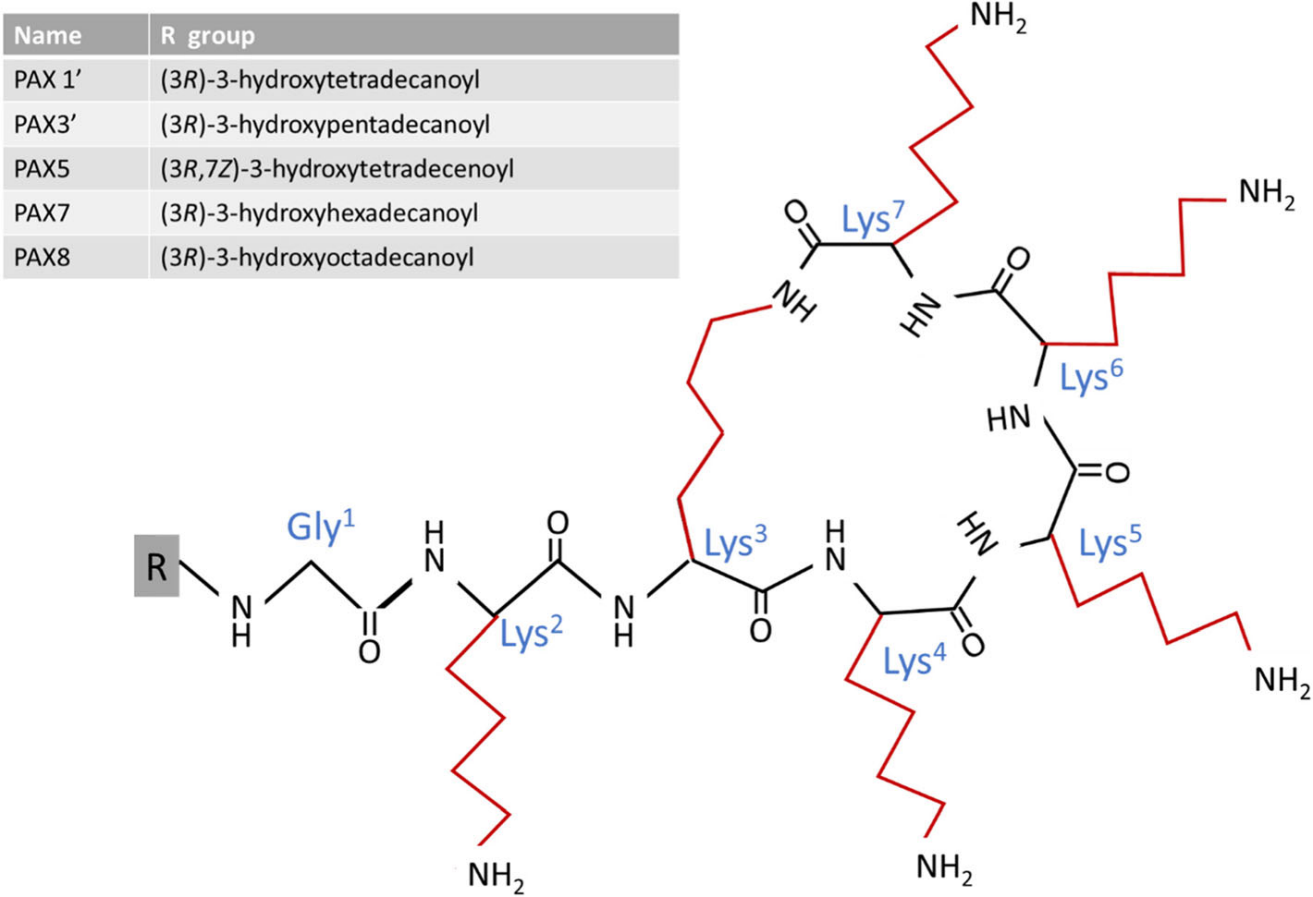
Primary structure of five known PAX lipopeptides [32] that were detected in the antimicrobial fractions of the *X. khoisanae* SB10 culture extracts

With the identification of the PAX1’ peptide there was a high probability of other PAX peptides in the HPLC fractions. In fraction C two more PAX peptides were found, namely a PAX peptide (*m/z* 1070.8091) which correlated to a linear analogue of PAX1’ (denoted PAX1’L) and a novel PAX peptide from the A group at *m/z* 1078.8119, that correlated to a PAX7 with an enoyl group in the lipid chain, denoted PAX7E1. This putative identification was done from the accurate *M*_r_, fragmentation pattern of the peptide moiety (refer to Additional file 1) and the fact that it eluted just before PAX7. This elution pattern correlated very well with that of PAX1’ and its 7-enoyl analogue PAX5 (see discussion below and Table 2). As there is already this identified 7-enoyl analogue in the PAX group, it is possible PAX7E1 is also a 7-enoyl analogue. We were, however, not able to confirm the structure of the R group with our in-analysis MS^e^ methodology, as the CID energy only released the lipid moiety and limited fragmentation was achieved. Fraction D contained PAX3’ (*m/z* 1066.8097) and fraction F contained PAX7 (*m/z* 1080.8286). This fraction also contained UV absorbing compounds (refer to Fig. 2) that are possibly the result of xenocoumacin breakdown (detected compounds with *m/z* 250.2162, 268.2263, 270.2425, 286.2369). The high PAX7 concentration in fraction F led to UPLC peak broadening, possibly due to the aggregation of this lipopeptide. Although PAX7 was the major compound in fraction F, this fraction also contained other PAX-like peptides. Two low abundance peptides with different elution times, but with the same *m/z* as PAX7E1 with a putative double bond (enoyl) in lipid chain, were observed at 3.71 and 3.88 min (*m/z* 1078.8096 and *m/z* 1078.8096, denoted PAX7E2 and PAX7E3). PAX7E2 displayed similar peptide chain fragments to that of PAX7 and PAX7E1, suggesting that the difference in elution time may or due to the position of the putative double bound in the lipid chain (refer to Additional file 1). PAX7E3 co-eluted with various compounds, so the fragmentation pattern was inconclusive although many similar Lys derived fragments were observed. Alternatively, it could be that PAX7E2 and PAX7E3 elutes later than PAX7E1 due to aggregation with PAX7 and other compounds, rather than structural differences. A peptide with *m/z* 1054.8090 co-eluted with the PAX7 peptide. From the accurate *M*_r_ it was derived that this peptide could be a linear PAX1’ without a hydroxyl-group (denoted PAX1’L-DH), but the structure remains unconfirmed due to the co-elution. Fraction G contained a number of PAX peptides, namely PAX5 (*m/z* 1050.7723), PAX1’ (*m/z* 1052.7936), PAX7 (*m/z* 1080.8280) and PAX8 (*m/z* 1106.8412). The fact that the same PAX peptides eluted in more than one fraction is possibly due to the formation of hetero-oligomers by the different lipopeptides, leading to elution at different acetonitrile concentrations during reverse phase chromatography. As in fraction D, fraction G also contained some UV absorbing compounds (refer to Fig. 2) that are possibly the result of xenocoumacin breakdown. Examples of the UPLC-MS chromatograms and spectra of the five most abundant PAX peptides are presented in Fig. 7. The primary structures of the known PAX peptides that were found in this study are given in Fig. 8. We were able to confirm the peptide sequence of most of the identified PAX peptides with our UPLC-MS^e^ procedure, except those that were found in very low concentrations. Similar fragment patterns to that depicted in Fig. 5 for PAX1’ were observed for the PAX peptides discussed above (refer to Additional file 1). This discovery and identification of the PAX lipopeptides were supported by the identification of the four modules coding for the PAX synthetase complex in the genome of *X. khoisanae* by our tblastx study (refer to Table 1).

## Conclusions

It was not surprising to discover the production of various antimicrobial compounds by *X. khoisanae* due to the *Xenorhabdus-Steinernema-*insect host tripartite interaction [1]. The known PAX peptides have only been isolated from *X. nematophila,* however, this is the first report of PAX peptides produced by *X. khoisanae.* PAX lipopeptides were first characterised by Gaulteri et al. [16] and to date 13 unique PAX peptides have been reported [34]. Our UPLC-MS and MS^e^ analyses and identification of PAX peptides and compounds in the antimicrobial complex of *X. khoisanae* SB10 have not been exhaustive, because of the complexity of many fractions containing labile compounds or unresolved compounds. There are certainly more antimicrobial compounds to discover in this strain’s natural antimicrobial complex in future studies, such as other xenocoumacins, xenorhabdins and compounds from the aryl polyene group, as indicated by the detection of the APE Ec biosynthetic complex (refer to Fig. 1). Because we consistently found the PAX lipopeptides and xenocoumacin in all the *X. khoisanae* SB10 culture extracts, we focused in this study on these compounds. Possible metabolites and degradation products related to the xenocoumacins were observed in some fractions (D and G), but after in-depth analysis of all the chromatographic fractions using UPLC-MS only one intact xenocoumacin, namely xenocoumacin 2, was identified in fraction B. It is possible that other xenocoumacins were also produced, but that the more labile compounds were lost during purification in a highly acidic environment. We were, however, able to identify five known Lys-rich lipopeptides from the PAX A-group with PAX1’ and PAX7 being the most abundant. We also discovered two putative linear PAX analogues, which could possibly be metabolic precursors of PAX1’. PAX7 could be related to the three unknown PAX peptides with the same *M*_r_ of 1077.80 but different retention on a reverse phase matrix. From their *M*_r_, fragmentation patterns and UPLC elution behaviour we putatively classified the three peptides as enoyl-derivatives of PAX7. We were, however, unable to confirm the R-group because of low abundance and co-elution, as well as limited fragmentation of the R-group in MS^e^ mode.

This study is the first to identify both the PAX peptides and a xenocoumacin in the antimicrobial complex of a *Xenorhabdus* species, as well as the first study on the antimicrobial complex of the Southern African *X. khoisanae* SB10. This report also highlights the natural tendency of *Xenorhabdus* species to produce antimicrobial complexes consisting of small antibiotics and AMPs. With the rising antibiotic resistance it may be wise to consider combining AMPs and small antibiotics, mimicking *Xenorhabdus*-type antimicrobial complexes.

## Methods

### Bacterial strains, growth media and growth conditions

*Xenorhabdus khoisanae* SB10 was maintained on NBTA [41], consisting of nutrient agar supplemented with bromothymol blue (0.025%, *w/v*) and TTC (0.004%, *w/v*). Incubation was at 30 °C. *Bacillus subtilis* subsp. *subtilis* BD170, *Escherichia coli* Xen 14 and *Candida albicans* CAB 392 were used as targets in the testing for antimicrobial activity. The bacteria and yeast were incubated at 37 °C. Bacteria were cultured on Brain Heart Infusion agar (Biolab Diagnostics, Gauteng, South Africa) and *C. albicans* on Potato Dextrose Agar (PDA, Biolab Diagnostics).

### Isolation of antimicrobial compounds

XAD-16 beads were activated by treating with 80% isopropanol containing 0.1% (*v/v*) TFA and added to TSB. After 30 min at 4 °C on an orbital shaker (100 rpm), the XAD-16 beads were removed and the medium autoclaved. *X. khoisanae* SB10 was inoculated into 5 ml untreated TSB and incubated at 26 °C for 24 h on a rotating wheel. The culture was added to 5 g activated XAD-16 beads, spread-plated onto XAD-16-treated TSB agar in petri dishes with a diameter of 135 mm and incubated at 26 °C for 96 h. Beads were collected from the plates and washed with sterile deionised water to remove the cells. Water was removed from beads by vacuum suction. The beads were washed with 150 mL 30% (*v/v*) ethanol for 15 min at 4 °C on an orbital shaker (100 rpm). Ethanol was removed by vacuum suction and the beads were washed with sterile deionised water. Amphipathic compounds were liberated from the beads, using 70% (*v/v*) isopropanol containing 0.1% (*v/v*) TFA (isopropanol-TFA). The eluent was filtered through a 0.45 μM cellulose nitrate filter and the isopropanol removed by using a rotary evaporator (RotaVapor® R-114, Büchi).

### Purification of antimicrobial compounds

The concentrated eluent was subjected to reverse phase chromatography on a 10 ml Sep-Pak C18 column (Waters, Milford, USA) on Perista Pump SJ-1211 (Chromatograph ATTO corporations, Tokyo, Japan). The column was washed with deionised water and compounds eluted by using a stepwise gradient ranging from 10 to 70% (*v/v*) isopropanol in analytical quality water with constant 0.1% TFA (*v/v*) in solvent system. The gradient was created with 10% increments per 10 min at 2 mL/minute flowrate. The fractions (denoted SPC fractions) were dried by rotary evaporation and tested for antimicrobial activity using the agar-well diffusion assay as described elsewhere.

Active SPC fractions were loaded onto a HiScale column (100 × 16 mm) packed with 15 RPC resin (GE Healthcare, South Africa) fitted to fast protein liquid chromatography (FPLC, ÄKTA purifier, GE Healthcare, South Africa). Fractions were eluted by a linear gradient of 10 to 55% B over 30 min, at a flow rate of 2.5 ml/min (A: analytical quality water containing 0.1%, v/v, TFA; B: HPLC grade acetonitrile containing 0.1%, *v/v*, TFA). Readings were recorded at 254 nm. Fractions were tested for antibacterial activity against *B. subtilis* subsp. *subtilis* BD170, using the agar-well diffusion assay as described elsewhere.

Fractions with antimicrobial activity collected from the ÄKTA purifier (FPLC active fractions) were lyophilized, resuspended in 50% (*v/v*) acetonitrile, loaded onto a Discovery BIO Wide Pore C_18_ HPLC column (10 μm, 250 × 10 mm; Sigma-Aldrich) and eluted by using a linear gradient (25 to 45%) of eluent B over 28 min at a flow rate of 2.0 ml/min. Further separation was on a Surveyor plus HPLC (Thermo Fisher Scientific, Waltham, Massachusetts, USA). Readings were recorded at 254 nm. Peak fractions were collected, dried and the antimicrobial activity tested as described elsewhere.

### Analysis of fractions with ultra-performance liquid chromatography and electrospray ionization mass spectrometry

Fractions with antimicrobial activity collected from the HPLC were subjected to UPLC, using an Aquity UPLC™ linked to a Waters Synapt G2 Mass Spectrometer (Waters Corporation, Miliford, USA). This was denoted UPLC-MS. Samples were prepared in 50% acetonitrile in water (1:1, *v/v*) at a concentration 200–1000 μg/mL. Samples were injected at 1–5 μl via Waters Acquity UPLC™ and chromatography was monitored in positive ESI mode and via PDA (230–400 nm). Separation of the components in each HPLC fraction were done on an Acquity UPLC® HSS T3 C_18_ column (1.8 μm particle size, 2.1 × 150 mm, Waters Corporation, Dublin, Ireland). Chromatography was done with analytical quality water modified with 0.1% (*v/v*) formic acid as eluent A and acetonitrile modified with 0.1% (*v/v*) formic acid as eluent B. The gradient developed at flow rate of 300 μl/min was as follows: 0 to 0.5 min at 40% B, linear gradient from 40 to 95% B from 0.5 to 11 min and 11 to 14 min at 95% B. The rest of the instrument settings for the UPLC-MS mode were as follows: cone voltage set at 15 V, a capillary voltage of 2.5 kV, cone voltage of 15 V, extraction cone voltage 4 V, source temperature of 120 °C, desolvation gas of 650 l/h and desolvation temperature of 275 °C. Data were collected in positive mode by scanning through *m/z* = 100 to 2000 in centroid mode at a rate of 0.2 scans/sec.

High resolution collisionally induced dissociation (CID) analyses were done in the MS^e^ mode (tandem MS or MS/MS) during the UPLC-MS and monitored on a second MS channel. CID were done at a collision energy gradient of 20 to 60 eV at 1 s MS/MS scan time. Data were collected in the second mass analyser (MS2) through *m/z* = 40 to 1500 in centroid mode. The rest of the instrument settings were as described above. To ensure reliable high-resolution MS data, the MS instruments were calibrated with sodium formate. Single point lock spray using leucine encephalin (*m/z* = 556.2771) as calibrant was used during analysis to compensate for any *m/z* drift.

### Antimicrobial activity of fractions from purification

SPC active fractions were suspended in analytical quality water, containing 0.1% (*v/v*) TFA to 350 mg/ml. The antimicrobial activity of fractions was tested using an agar-well diffusion assay in a micro well titre plate. In short, the appropriate growth media containing 1.0% (*w/v*) agar was seeded with a dense 12-h-old culture of *B. subtilis* subsp. *subtilis* BD170, *E. coli* Xen 14 (1.0%, *v/v*) or *C. albicans* CAB 392 (1.0%, *v/v*). Wells were made into the agar and 15 μL of each fraction dispensed into a well. Plates were incubated for 24 to 48 h at 37 °C. A clear zone surrounding the well indicated activity. Analytical quality water, containing 0.1% (v/v) TFA, was used as negative control. Ciprofloxacin was used as positive control for *B. subtilis* subsp. *subtilis* BD170 and *E. coli* Xen 14 and amphotericin B for *C. albicans* CAB 392.

### Temperature stability

SPC active fractions of 350 mg/mL were prepared in MilliQ water, containing 0.1% (v/v) TFA. The suspension was autoclaved for 20 min and tested for antimicrobial activity against *B. subtilis* subsp. *subtilis* BD170, using the agar-well diffusion assay as described elsewhere. Plates were incubated at 37 °C for 24 h. The diameter of growth inhibition zones was recorded and compared to controls. This was done by using the software program ImageJ (v. 1.48).

## Additional file

Additional file 1: Detailed mass spectrometric analysis of the chromatographic fractions of *X. khoisanae* extracts. (PDF 471 kb)

## Data Availability

The datasets generated and/or analysed during the current study are not publicly available due to the preparation of a patent, but are available from the corresponding author (LMTD) on reasonable request.

## Abbreviations

FPLC: Fast protein liquid chromatography
HPLC: High pressure liquid chromatography
MS: Mass spectrometer/mass spectrometry
NRPS: Non-ribosomal peptide synthetase
PAX: Peptide-antimicrobial-*Xenorhabdus*
TFA: Trifluoroacetic acid
TSB: Tryptic soy broth
TTC: Triphenyl tetrazolium chloride
UPLC: Ultraperformance liquid chromatography
