# Supplementary material for: Xenorhabdus khoisanae SB10 produces Lys-rich PAX lipopeptides and a Xenocoumacin in its antimicrobial complex
Source: BMC Microbiol. 2019 Jun 13;19:132. doi: 10.1186/s12866-019-1503-x (PMC6567599; doi:10.1186/s12866-019-1503-x)
Supplement: Supplementary file 1 — Detailed mass spectrometric analysis of the chromatographic fractions of X. khoisanae extracts. (PDF 471 kb) [file 12866_2019_1503_MOESM1_ESM.pdf]

SUPPLEMENTARY DATA

Xenorhabdus khoisanae SB10 produces Lys-rich PAX lipopeptides and a Xenocoumacin in its antimicrobial complex

J. Dreyer[1], M. Rautenbach[2*], E. Booysen[1], A.D. van Staden[1], S.M. Deane[1] and L.M.T. Dicks[1*]

[1]Department of Microbiology and [2] BIOPEP Peptide Group, Department of Biochemistry, Stellenbosch University, Private Bag X1, 7602 Matieland, South Africa

Detailed mass spectrometric analysis of the chromatographic fractions of *X. khoisanae* extracts

The detailed UPLC-MS profiles of the chromatography of the HPLC fractions B, C, D, F and G is given in **A** of the following blocked figures. The top profile shows the UPLC-MS^e of the fraction with monitoring for the Lys fragment at *m/z* =129.10. This monitored UPLC profile indicates all the possible PAX peptides in a fraction. The bottom chromatogram is the UPLC-MS of the fraction with monitoring for a specific PAX peptide.

The ESMS spectrum of the combined peak of the specific PAX peptide (with front and back section subtracted) is shown in **B** for each of the PAX peptides given in Table 1. This high resolution ESMS spectrum shows the typical $[M+H]^+$, $[M+2H]^{2+}$ and $[M+3H]^{3+}$ molecular ions for all the detected PAX peptides. The main PAX peptides also exhibited a strong $[M+4H]^{4+}$.

The CID spectrum of the selected PAX peptide is shown in **C** of each blocked figure. Only fragment peaks at >10% signal are shown. In order to limit interference of fragments from co-eluting contaminants a narrow spectral range with the highest peptide purity in the peak were used for fragment analysis. All the peptides show a typical fragmentation pattern of a Lys-rich peptide with a neutral loss of 128.09 from the major fragments. The majority of ions with a neutral fragment loss, as well as most of the intact peptide ions, had resultant dehydration product ion that is related to the Lys residues in the peptide. Not all the expected product or fragment ions were detected above 10% signal intensity, and this was especially problematic for the PAX peptides at low concentration. Lys-derived fragment ions with *m/z* 129.1015 (Lys residue ion) and 84.0799 (Lys immonium ion) were observed for all the PAX peptides.

Fraction B, PAX1’ at R_t 2.93 min

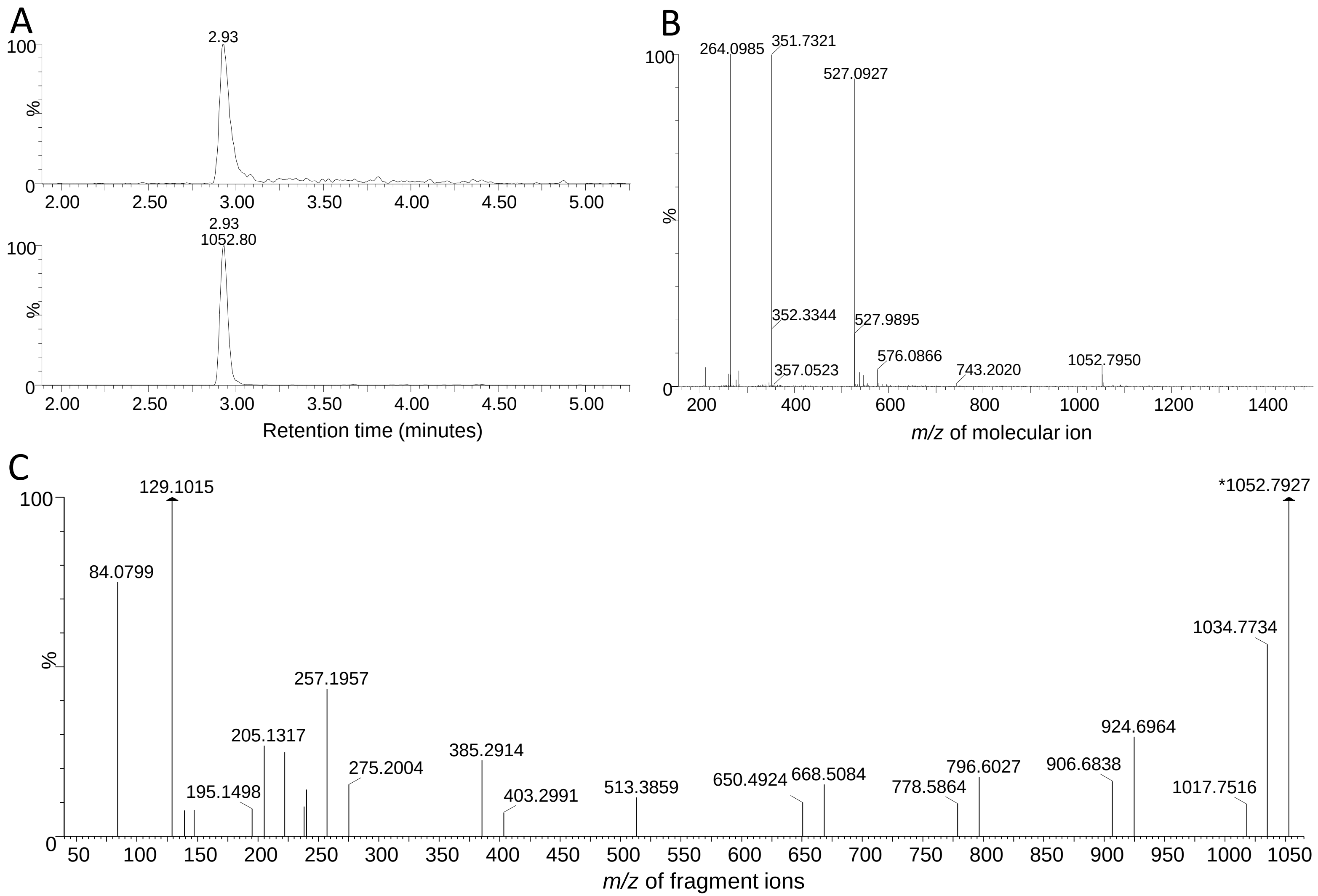

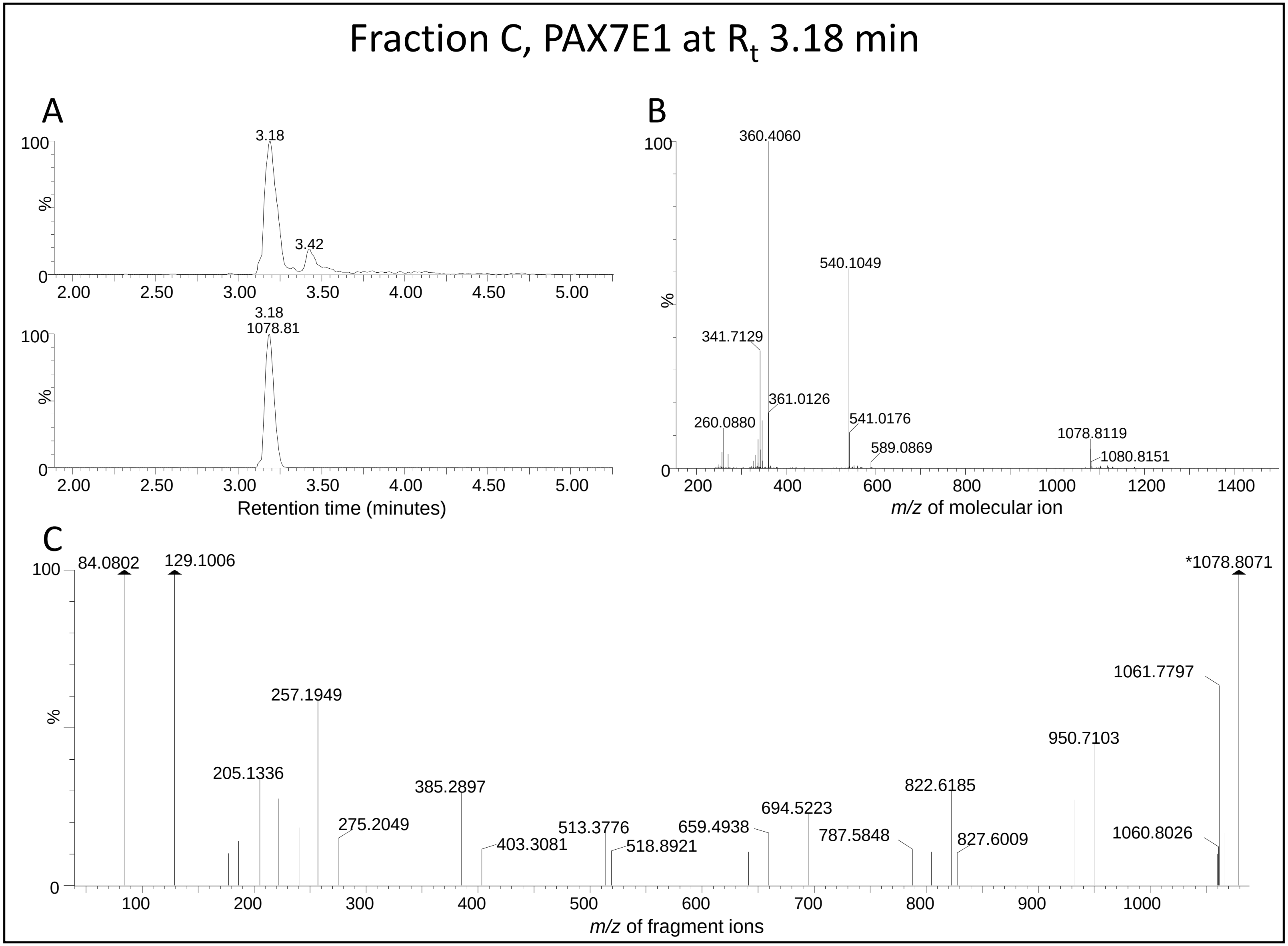
Fraction C, PAX7E1 at R_t 3.18 min
A
3.18
3.42
3.18
1078.81
Retention time (minutes)
B
360.4060
540.1049
341.7129
361.0126
541.0176
260.0880
589.0869
1078.8119
1080.8151
m/z of molecular ion
C
84.0802
129.1006
*1078.8071
1061.7797
257.1949
950.7103
205.1336
385.2897
822.6185
694.5223
275.2049
513.3776
659.4938
787.5848
1060.8026
827.6009
403.3081
518.8921
m/z of fragment ions

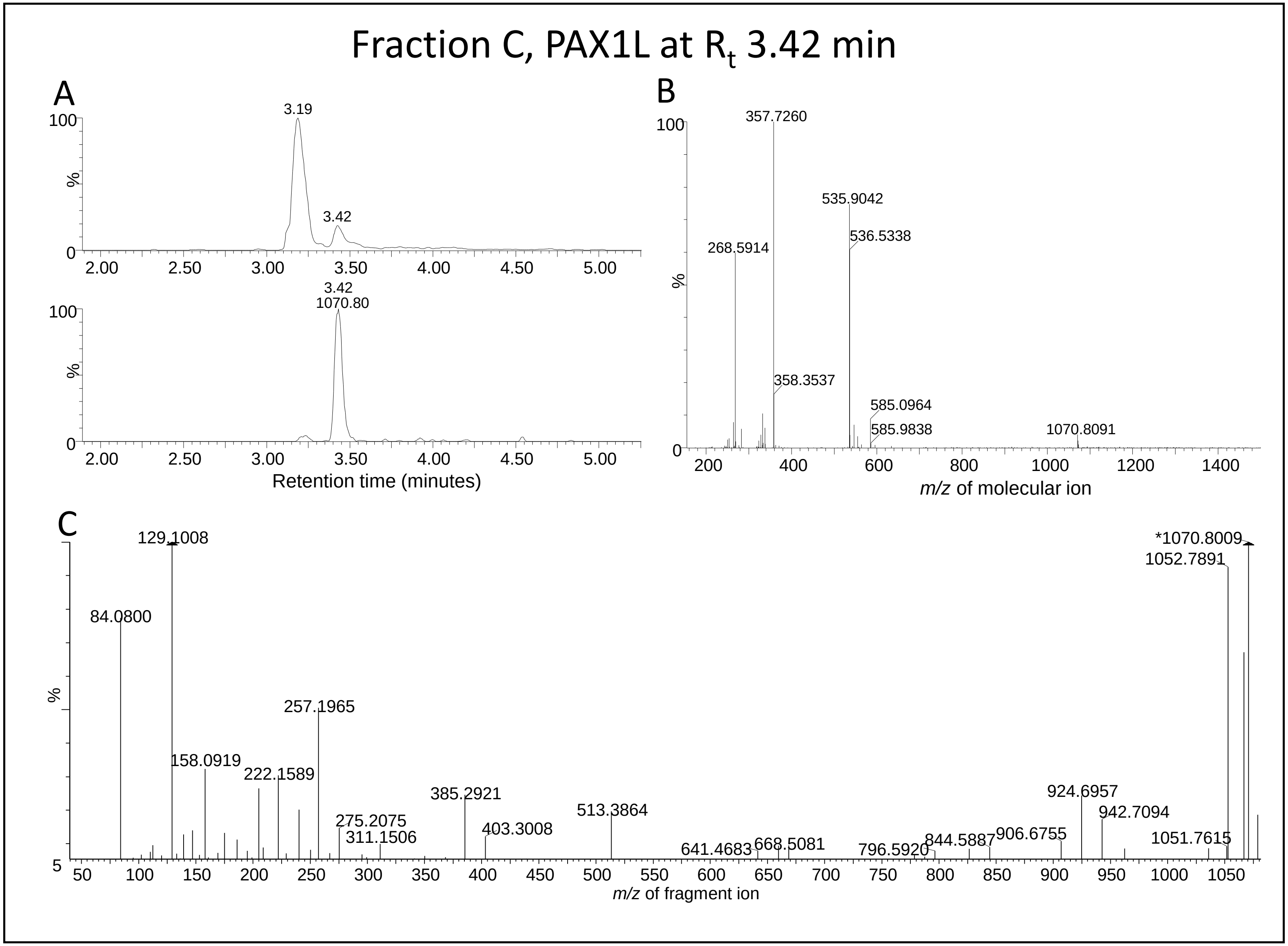
Fraction C, PAX1L at Rt 3.42 min
A
3.19
3.42
100
%
0
2.00 2.50 3.00 3.50 4.00 4.50 5.00
3.42
1070.80
Retention time (minutes)
B
357.7260
535.9042
536.5338
268.5914
358.3537
585.0964
585.9838
1070.8091
200 400 600 800 1000 1200 1400
m/z of molecular ion
C
129.1008
*1070.8009
1052.7891
84.0800
257.1965
158.0919
222.1589
385.2921
513.3864
275.2075
311.1506
403.3008
641.4683
668.5081
796.5920
844.5887
906.6755
924.6957
942.7094
1051.7615
5
50 100 150 200 250 300 350 400 450 500 550 600 650 700 750 800 850 900 950 1000 1050
m/z of fragment ion

Fraction D, PAX3’ at R_t 3.29 min

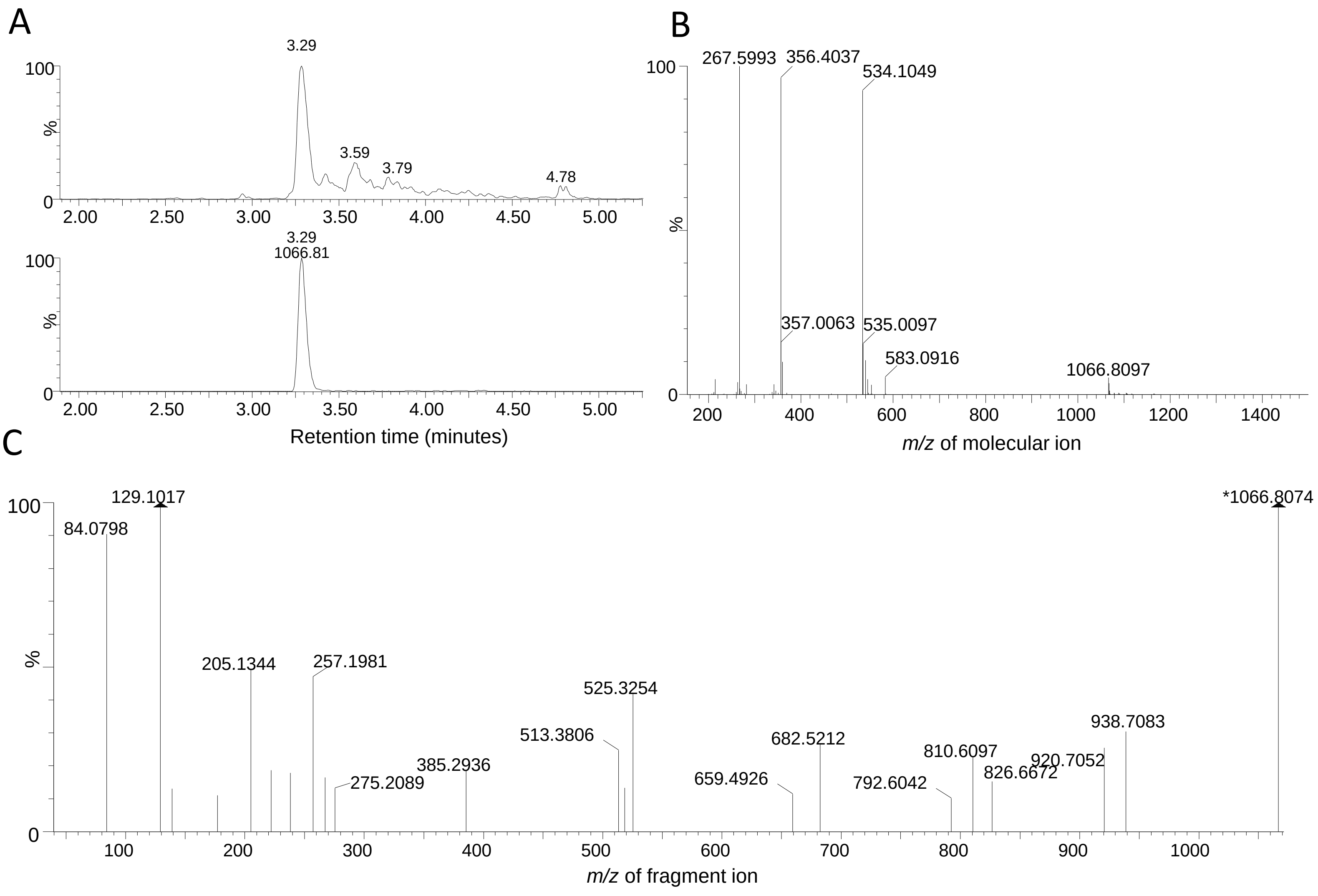

Fraction F, PAX1L-DH at R_t 3.47 min

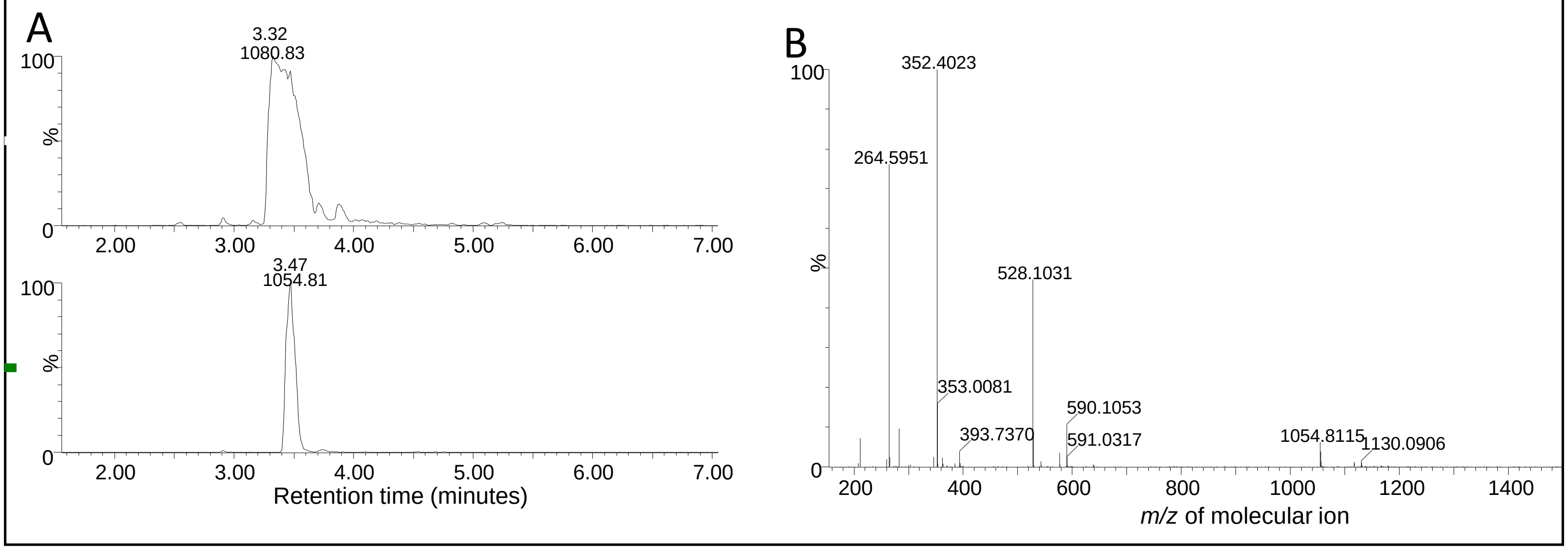

Fraction F, PAX7 at R_t 3.32-3.47 min

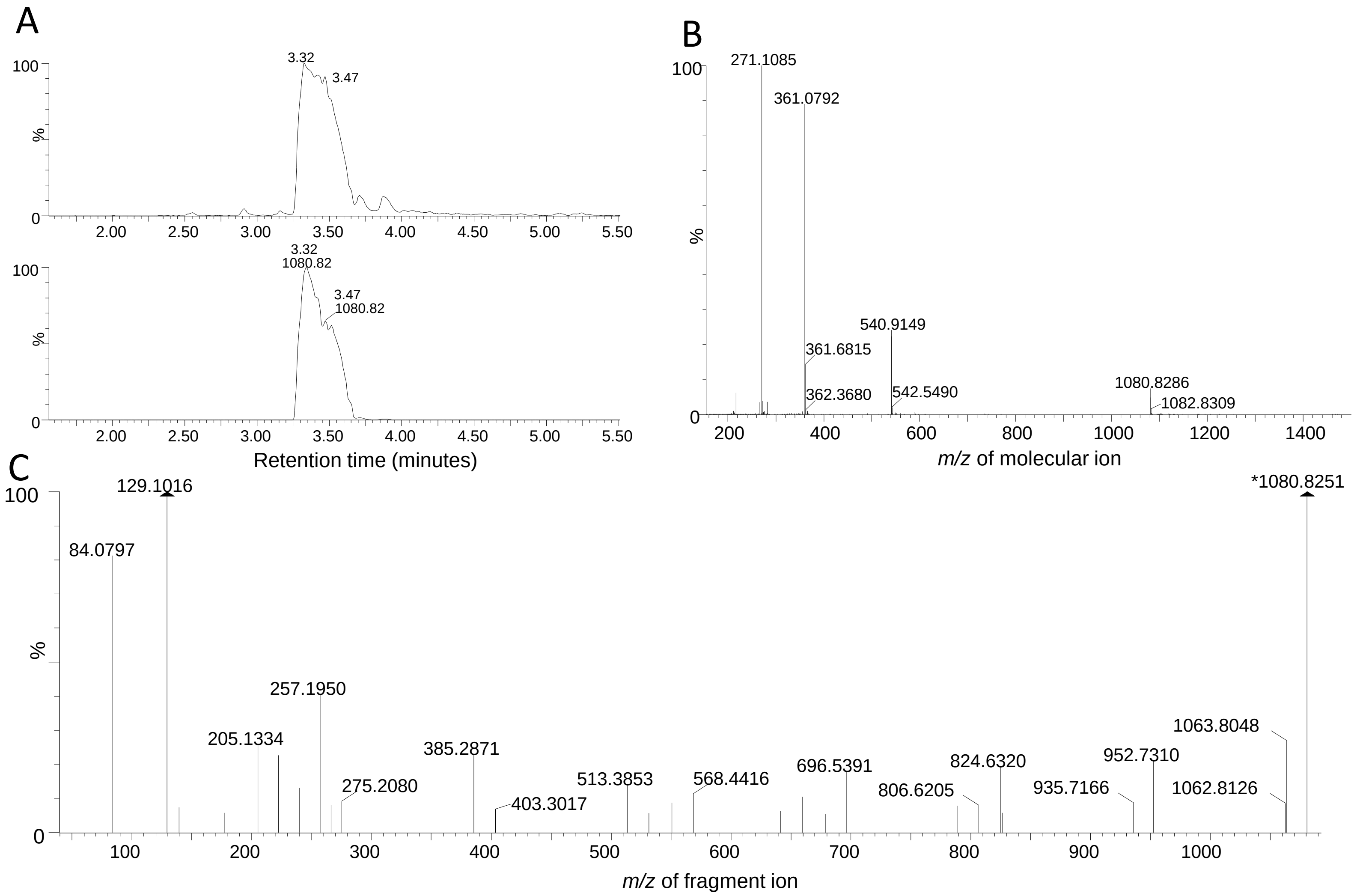

Fraction F, PAX7E2 and PAX7E3 at R_t 3.71 and 3.88 min

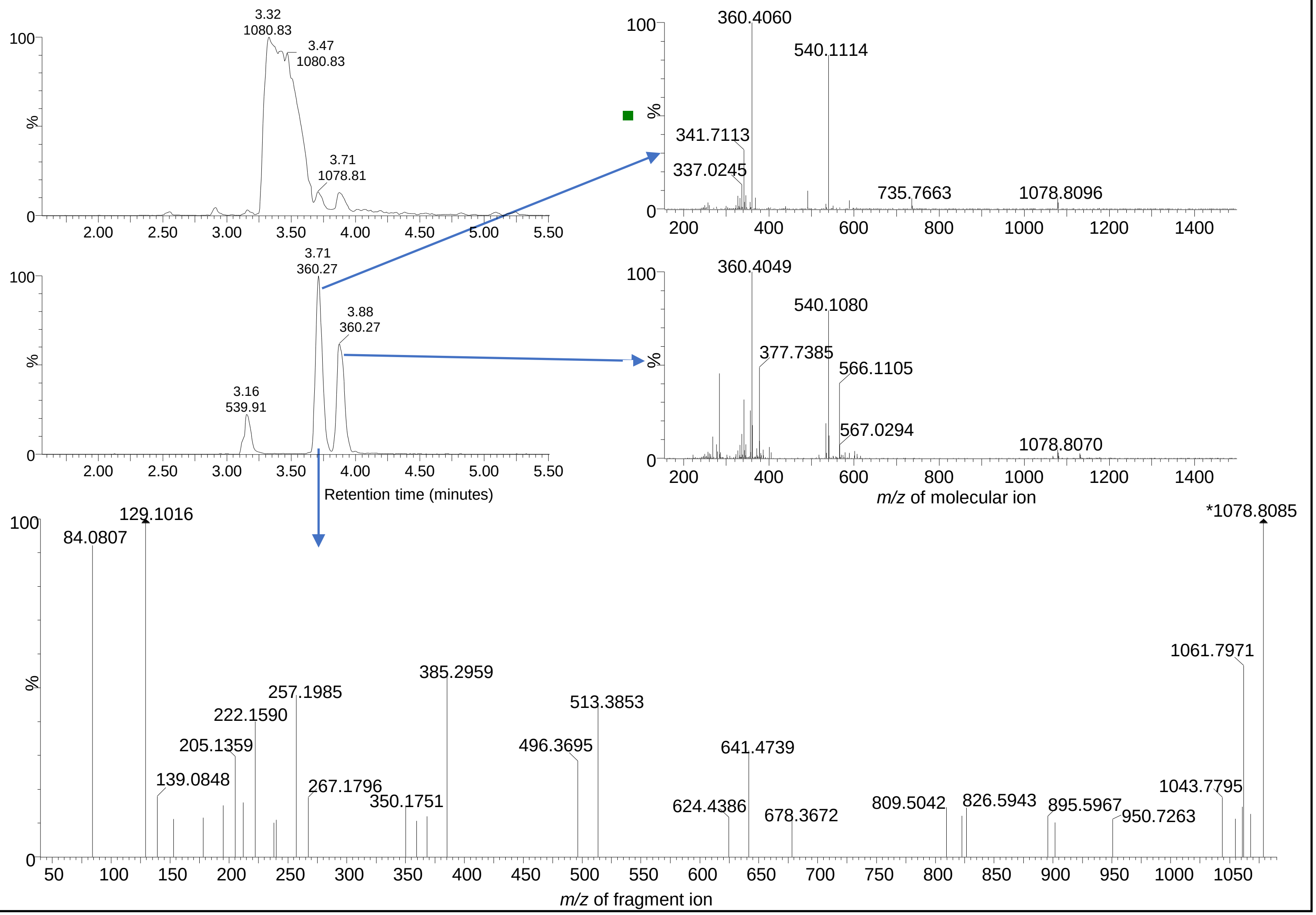

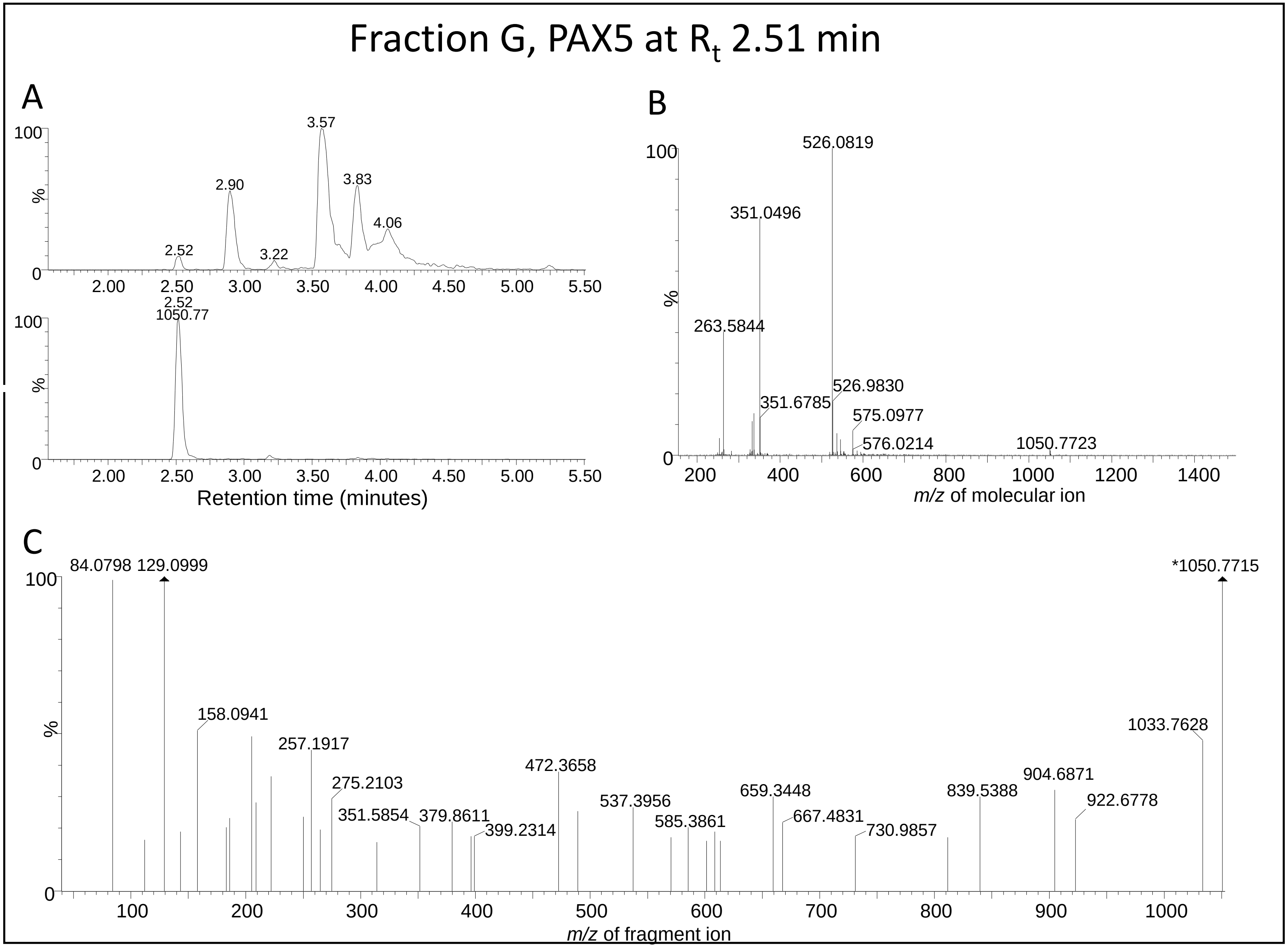

Fraction G, PAX5 at R_t 2.51 min
A
100
%
0
2.52
2.90
3.22
3.57
3.83
4.06
2.00
2.50
3.00
3.50
4.00
4.50
5.00
5.50
2.52
1050.77
Retention time (minutes)
B
100
%
0
526.0819
351.0496
263.5844
526.9830
351.6785
575.0977
576.0214
1050.7723
200
400
600
800
1000
1200
1400
m/z of molecular ion
C
100
%
0
84.0798
129.0999
*1050.7715
158.0941
257.1917
275.2103
351.5854
379.8611
399.2314
472.3658
537.3956
585.3861
659.3448
667.4831
730.9857
839.5388
904.6871
922.6778
1033.7628
100
200
300
400
500
600
700
800
900
1000
m/z of fragment ion

Fraction G, PAX1' at R_t 2.90 min

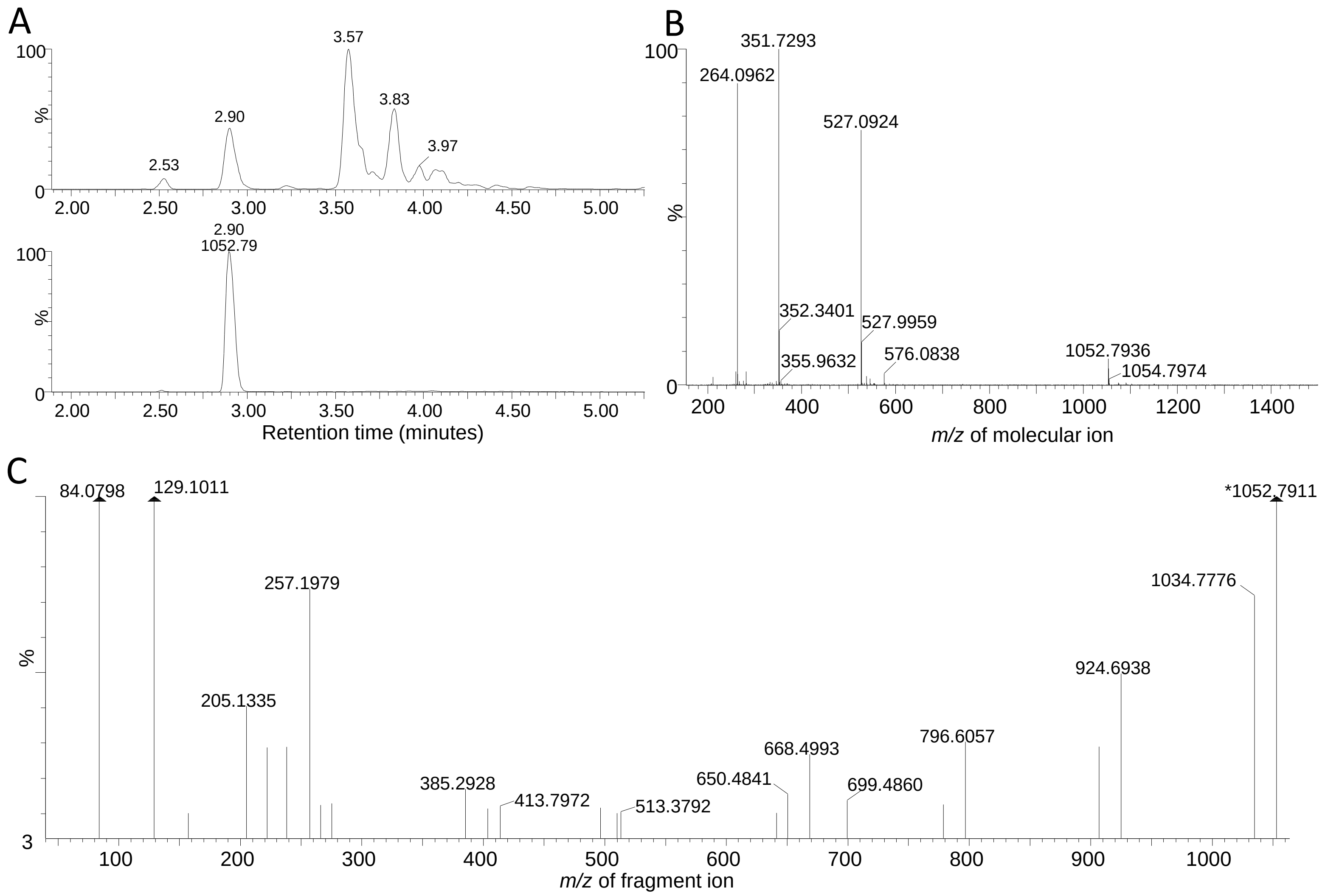

Fraction G, PAX7 at R_t 3.57 min

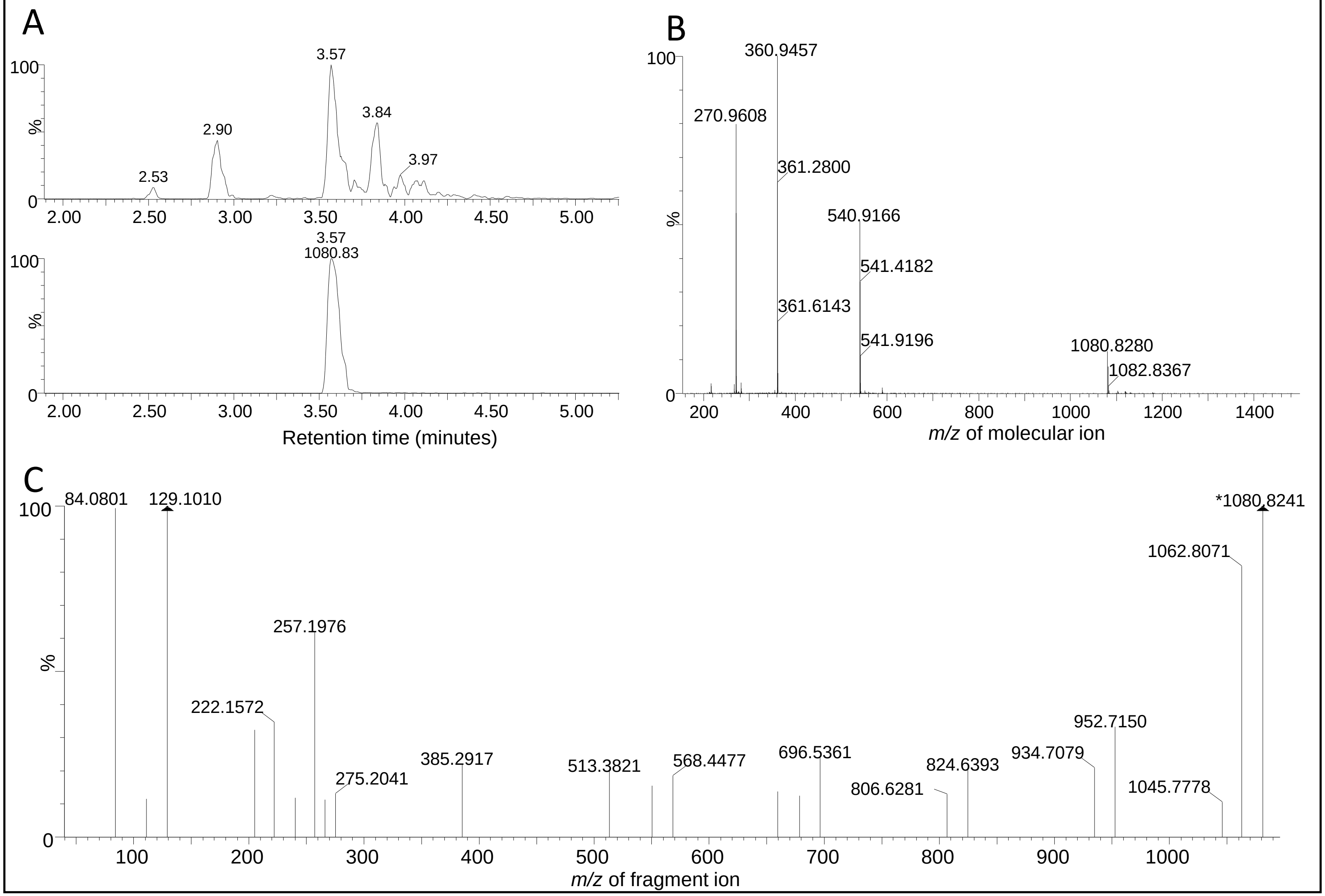

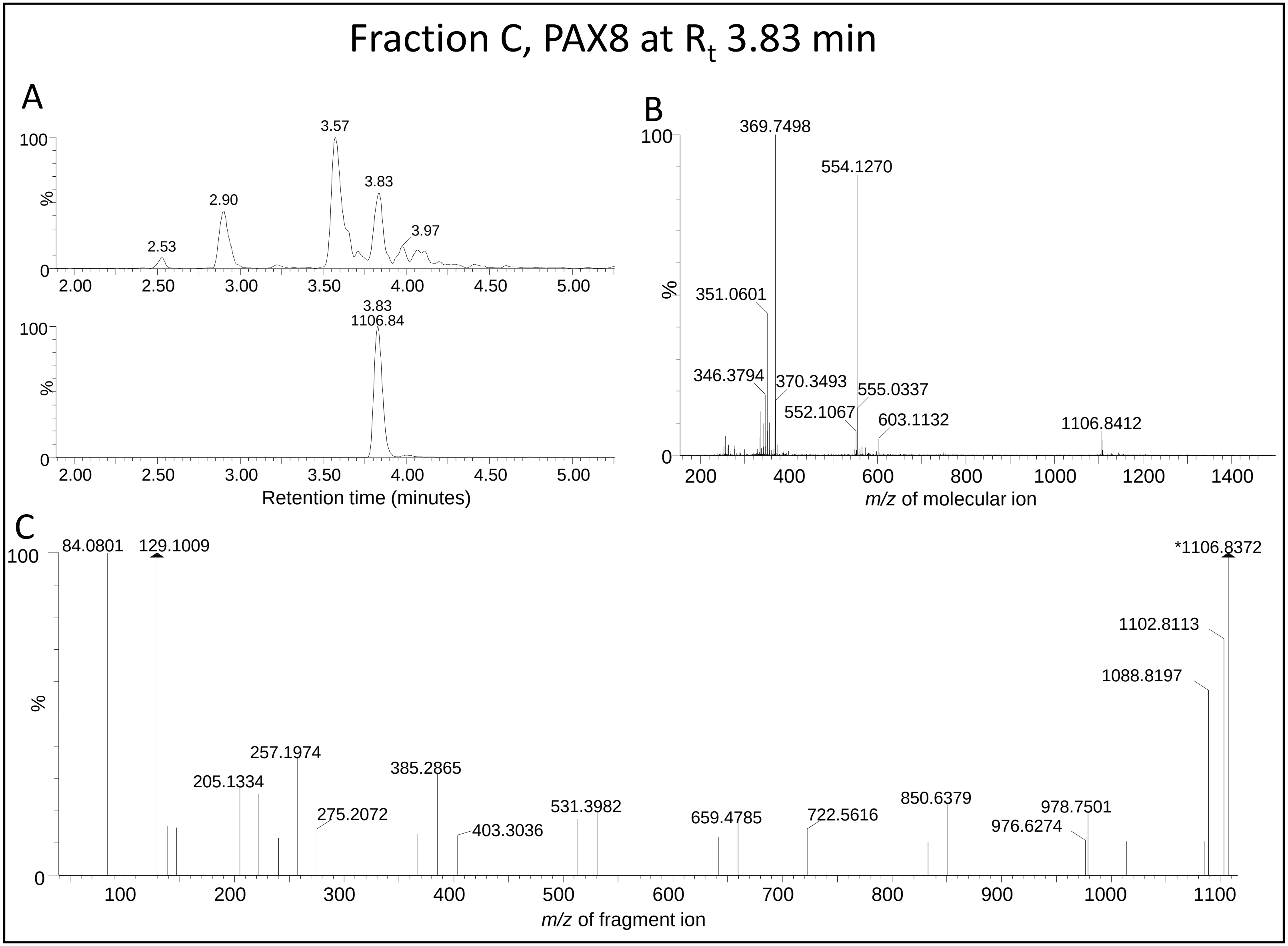
Fraction C, PAX8 at R_t 3.83 min
A
100
%
0
2.53
2.90
3.57
3.83
3.97
2.00
2.50
3.00
3.50
4.00
4.50
5.00
3.83
1106.84
Retention time (minutes)
B
369.7498
554.1270
351.0601
346.3794
370.3493
555.0337
552.1067
603.1132
1106.8412
200
400
600
800
1000
1200
1400
m/z of molecular ion
C
84.0801
129.1009
*1106.8372
1102.8113
1088.8197
257.1974
205.1334
385.2865
275.2072
403.3036
531.3982
659.4785
722.5616
850.6379
978.7501
976.6274
100
200
300
400
500
600
700
800
900
1000
1100
m/z of fragment ion
